# A Novel *KCNH2* S981fs Mutation Identified by Whole-Exome Sequencing Is Associated with Type 2 Long QT Syndrome

**DOI:** 10.3390/ijms241612742

**Published:** 2023-08-13

**Authors:** Yu-Wen Cheng, Chia-Tung Wu, Chi-Jen Chang, Yung-Hsin Yeh, Gwo-Jyh Chang, Hsin-Yi Tsai, Lung-An Hsu

**Affiliations:** 1 Cardiovascular Division, Chang Gung Memorial Hospital, Chang Gung University College of Medicine, Tao-Yuan 33305, Taiwan; b9702089@cgmh.org.tw (Y.-W.C.); doghead.wu@gmail.com (C.-T.W.); chijenformosa@gmail.com (C.-J.C.); yys0tw@yahoo.ca (Y.-H.Y.); sunny66house@yahoo.com.tw (H.-Y.T.); 2 Graduate Institute of Clinical Medical Sciences, Chang Gung University, Tao-Yuan 33305, Taiwan; gjchang@mail.cgu.edu.tw

**Keywords:** long QT syndrome, exome sequencing, KCNH2, mutation

## Abstract

*KCNH2* loss-of-function mutations cause long QT syndrome type 2 (LQT2), an inherited cardiac disorder associated with life-threatening ventricular arrhythmia. Through whole-exome sequencing, we discovered a novel AGCGACAC deletion (S981fs) in the *hERG* gene of an LQT2 patient. Using a heterologous expression system and patch clamping, we found that the mutant K channel had reduced cell surface expression and lower current amplitude compared to the wild type. However, functional expression was restored by lowering temperature and using potassium channel inhibitors or openers (E4031, cisapride, nicorandil). Co-immunoprecipitation experiments confirmed the assembly of mutant proteins with wild-type hERG. Confocal imaging showed decreased hERG distribution on the cell membrane in cells expressing S981fs. Notably, treatment with G418 significantly increased hERG current in wild-type/S981fs heterozygotes. In conclusion, our study identifies a novel *hERG* mutation leading to impaired Kv11.1 function due to trafficking and nonsense-mediated RNA decay defects. These findings shed light on the mechanisms underlying LQT2 and offer potential therapeutic avenues.

## 1. Introduction

Long QT syndrome (LQTS) is a genetic cardiac disorder characterized by prolonged QT intervals on electrocardiography, which can result in life-threatening ventricular arrhythmias, syncope, or sudden cardiac death. LQT2 is predominantly caused by loss-of-function mutations in the alpha subunit of the rapidly activating delayed rectifier potassium channel (Kv 11.1), which is encoded by the human ether-à-go-go-related gene (*hERG*). These mutations lead to a reduction in repolarizing current, contributing to the pathogenesis of LQT2 [1].

In LQT2, *hERG* mutations can be classified into different mechanistic categories. Approximately 40% of the mutations are nonsense mutations that trigger nonsense-mediated RNA decay (NMD), resulting in haploinsufficiency [2,3]. The remaining 60% consist of missense mutations, which can lead to trafficking-deficient channel proteins (class 2 mechanism), abnormal channel gating (class 3 mutation), or altered channel selectivity or permeability (class 4 mutation) [2,4,5]. Among these, trafficking deficiency is the most common cause of missense mutations, with approximately two-thirds of the mutations exhibiting this mechanism [2,6]. Additionally, missense mutations can demonstrate either haploinsufficiency or a dominant-negative effect, with the latter associated with a worse clinical outcome [3].

In our study, we identified a novel eight-base-pair (bp) AGCGACAC deletion within exon 12 of the *hERG* gene at nucleotide positions 2941 and 2948 [c.2941-2948AGCGACAC] in a Taiwanese patient with LQT2. This deletion induces a frameshift and premature termination of the protein (p.S981fs). Based on the predicted consequences, this mutation is expected to impair Kv11.1 function due to a combination of trafficking impairment and NMD. At the cellular level, we observed that the deficiency could be partially corrected by the administration of the potassium channel blocker/opener and antibiotic G418.

## 2. Results

### 2.1. Functional Validation of the Novel Mutation Identified by Exome Sequencing

**Figure 1:**
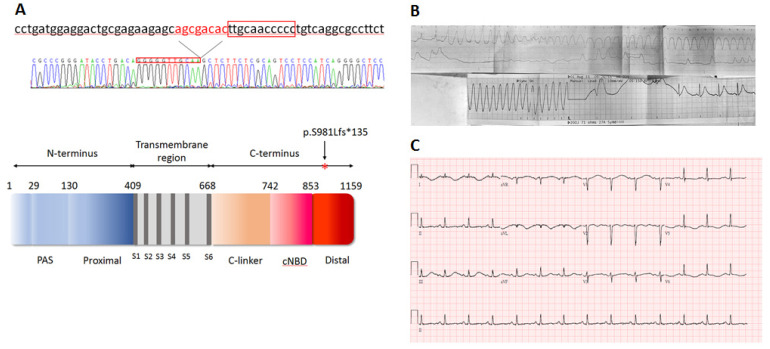
(**A**) (Upper half) Sequencing result (reverse sequence chromatogram) showing the *KCNH2* c.2941-2948 AGCGACAC 8 bp deletion mutation (S981Lfs*135). (Lower half) Schematic of the hERG protein important domains and the location of frameshift mutation S981Lfs*135. (**B**) Electrocardiogram (ECG) strip recording of the proband during her emergency room visit, displaying torsades de pointes and ventricular tachycardia before and after cardioversion. (**C**) Twelve-lead ECG of the proband after cardioversion, demonstrating a prolonged QT interval.

**Figure 2:**
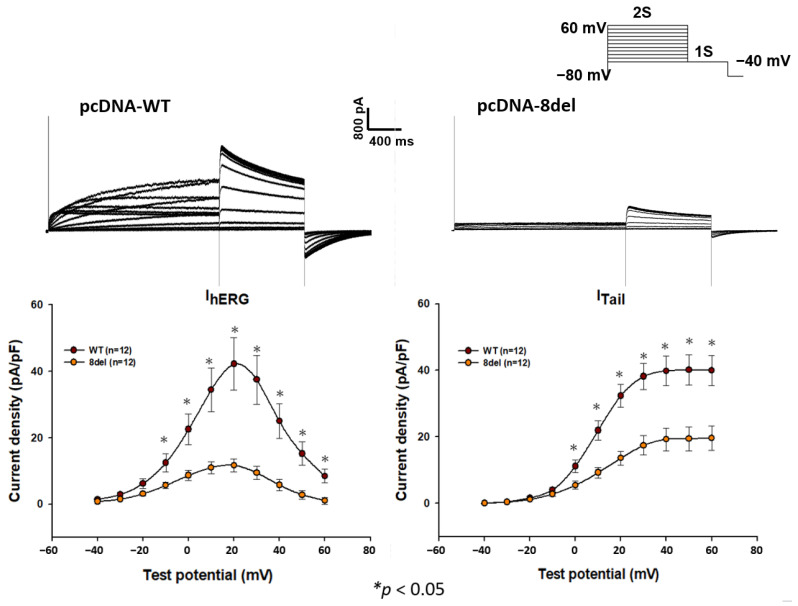
(Upper half) The current densities of HEK293T cells transfected with wild type (WT) and S981fs (8del). (Lower half) Quantification of step currents and tail currents for WT and S981fs hERG channels. The tail currents were normalized with Boltzmann equation to fit with the average data. *, *p* < 0.05.

The whole-exome sequencing (WES) analysis revealed a heterozygous frameshift mutation (S981fs) in the *KCNH2* gene of the proband. Figure 1 illustrates the location of the eight bp AGCGACAC deletion and the resulting frameshift mutation within the DNA sequence and the hERG protein. The identified mutation in our study is considered novel, as it has not been previously reported in the Taiwan Biobank and the Genome Aggregation Database (gnomAD). Since parental DNA analysis was not conducted, we were unable to ascertain whether this mutation is a de novo occurrence or not. To assess the functional consequences of the mutation, we conducted whole-cell patch clamp recordings on HEK293 cells transfected with wild-type or mutant hERG cDNA. The recordings, presented in Figure 2, demonstrated that the wild-type hERG channels exhibited typical hERG current with voltage-dependent activation. In contrast, the S981fs mutant hERG channels generated currents with significantly reduced amplitude. The mean peak tail-current density of the S981fs channel was significantly lower than that of the wild-type channel (19.65 ± 3.71 pA/pF, *n* = 12, vs. 39.96 ± 4.59 pA/pF, *n* = 12; *p* < 0.05).

### 2.2. Protein Processing and Traffiking of the Mutant Protein

**Figure 3:**
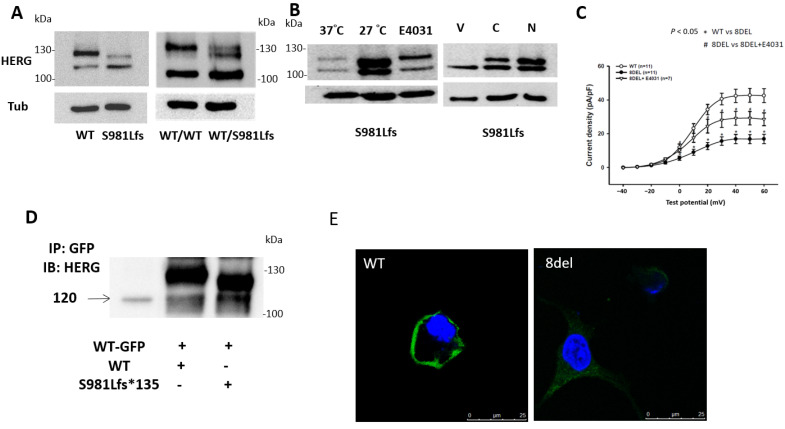
(**A**) Western blot analysis comparing wild-type (WT) and S981Lfs hERG proteins. The upper band (155 kDa) represents mature hERG, while the lower band (135 kDa) corresponds to immature hERG. Alpha tubulin (Tub) serves as a control. (**B**) HEK293T cells transfected with S981Lfs were incubated under different conditions, including low temperature (27 °C), E4031, Vehicle (V), Cisapride (C), or Nicorandil (N), resulting in increased expression of both mature and immature hERG channels. (**C**) Current–voltage relationship of HEK293T cells transfected with WT and S981Lfs (8del), incubated with or without E4031; * *p* < 0.05, ^#^ *p* < 0.05. (**D**) Co-transfection of WT-HERG-GFP and untagged WT- or S981Lfs-HERG constructs in HEK293T cells. Immunoprecipitation (IP) with anti-GFP antibody followed by Western blotting (IB) using anti-hERG antibody revealed the presence of both WT and S981Lfs proteins. (**E**) Confocal imaging of non-permeabilized HEK293T cells transfected with either WT or S981fs proteins (without GFP tag), incubated with anti-hERG antibody against extracellular peptide. The images demonstrate detectable expression of WT and S981fs hERG on the cell membranes, with weaker fluorescence observed on the membranes of S981fs-transfected cells. Green signals indicate hERG, and blue signals indicate nuclei.

The *KCNH2* gene undergoes transcription, splicing, and translation to produce the Kv11.1a α-subunit, which is initially core-glycosylated with a molecular mass of 135 kDa. It undergoes further glycosylation in the Golgi apparatus, resulting in an increased molecular mass of 155 kDa. To examine the impact of the S981fs mutation on protein processing, we performed Western blot analysis on whole-cell lysates. Figure 3A displays the Western blot analysis after mono- or co-transfection of wild type and S981fs-hERG into HEK293T cells. The results showed two bands of protein, with the S981fs-hERG allele exhibiting a slightly smaller size than the wild-type allele. Both mono- and co-transfection studies revealed weaker Western blot signals for the mature Kv11.1 protein band compared to the immature band in the S981fs mutant. This suggests that the S981fs mutation may partially reduce hERG current through a trafficking deficiency mechanism.

Based on previous research [2], it has been observed that some LQT2-Kv11.1 channels can undergo improved Golgi processing and surface membrane expression under reduced temperature culture conditions or when exposed to IKv11.1-blocking drugs like E4031. Therefore, we investigated whether protein processing could be rescued in S981fs-Kv11.1 channels through temperature or pharmacological correction. Figure 3B presents representative Western blot analyses of cells expressing S981fs-Kv11.1 channels cultured under different conditions, including control conditions (37 °C), at 27 °C (for 24 h), or in the presence of E4031 (10 μM), cisapride (100 nM), or nicorandil (100 nM) for 24 h. The results showed that protein processing of S981fs-Kv11.1 channels was corrected in cells incubated at 27 °C or treated with E4031, cisapride, or nicorandil. To further assess the functional activity of S981fs-Kv11.1 channels, cells transfected with S981fs were incubated with culture medium containing E4031. As shown in Figure 3C, cells transfected with S981fs alone exhibited significantly lower currents compared to wild-type cells, while cells transfected with S981fs and incubated with E4031 displayed a current density (28.55 ± 3.78 pA/pF, *n* = 7, *p* < 0.05) higher than that of cells transfected with S981fs alone (16.90 ± 2.73 pA/pF, *n* = 11) but still lower than that of cells transfected with the wild-type protein (42.55 ± 4.15 pA/pF, *n* = 11).

To investigate the interaction between mutant and wild-type proteins, we conducted co-immunoprecipitation experiments in HEK293T cells. Figure 3D demonstrates that both wild-type and S981fs proteins were detectable on Western blotting, indicating that the mutant proteins were capable of interacting with wild-type hERG-GFP and did not exhibit any assembly defects. Additionally, non-permeabilized HEK293T cells transfected with wild-type or S981fs proteins (without GFP tagging) were incubated with an anti-hERG antibody against the extracellular peptide. Confocal images in Figure 3E revealed the detectability of both proteins on the cell membranes of HEK293T cells. However, the green fluorescence on the membranes of S981fs cells was weaker than that on the membranes of wild-type cells, indicating decreased membrane expression of mutant hERG channels. These findings further support the conclusion that the S981fs mutation exhibits partial trafficking deficiency.

### 2.3. Possibility of Nonsense-Mediated RNA Decay (NMD)

**Figure 4:**
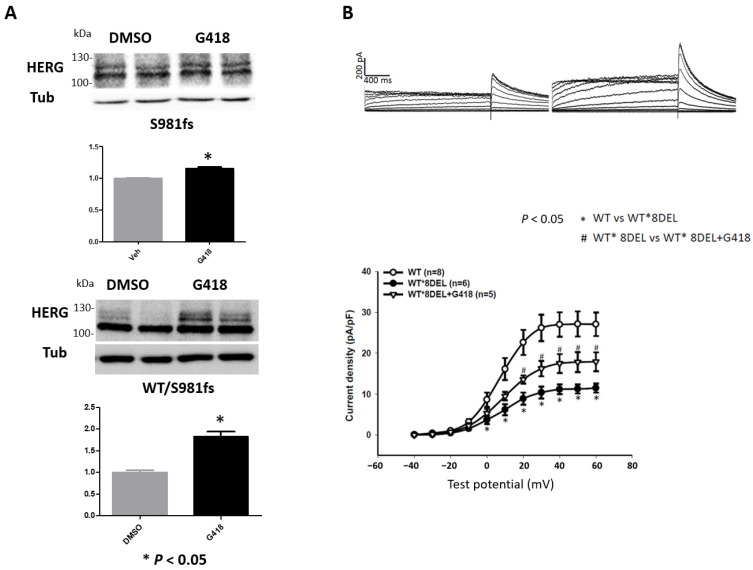
(**A**) Western blot of HEK293T cells mono-transfected with S981fs (upper half) and co-transfected with wild-type (WT) and S981fs hERG (WT/S981fs) (lower half) pre-treated with or without G418 (using DMSO as a vehicle). The upper band (155 kDa) was recognized as mature hERG, and the lower band (135 kDa) was shown as immature hERG. Alpha tubulin (Tub) was detected as control. The relative expression level of hERG protein was quantified by densitometry and normalized to the control level, which was set at 1.0. Each value represents mean ± SE of more than three independent experiments. (**B**) HERG current traces and current-voltage relationship of cells co-transfected with WT and S981fs hERG (WT*8DEL) incubated with or without G418; * *p* < 0.05, ^#^ *p* < 0.05.

Considering the predicted damaging effect of S981fs through NMD, we investigated the impact of aminoglycoside antibiotics G418 on the S981fs mutant proteins. The results, presented in Figure 4A, show that incubating HEK293T cells transfected with S981fs alone or co-transfected with S981fs and wild-type hERG with culture medium pre-treated with G418 significantly enhanced the expression of S981fs mutant proteins. Figure 4B displays the hERG currents of wild-type, wild-type/S981fs, and wild-type/S981fs cells treated with G418. The peak tail-current density of wild type/S981fs was significantly improved after G418 treatment (11.49 ± 1.11 pA/pF, *n* = 6; vs. 17.91 ± 2.31 pA/pF, *n* = 5; *p* < 0.05). The tail current of wild type/S981fs is lower than 50% of the tail current produced by wild-type cells (27.07 ± 2.86 pA/pF, *n* = 8), suggesting a dominant-negative effect. However, the improvement in IKr activity through G418 treatment was not statistically significant in S981fs mono-transfection.

## 3. Discussion

Long QT syndrome (LQTS) is a condition that can lead to life-threatening arrhythmias and sudden cardiac death. In our study, we have identified a novel mutation in the *hERG* gene which is responsible for encoding the IKr ion channel. This mutation causes a frameshift effect in the Kv 11.1 alpha subunit protein after the cyclic nucleotide-binding domain (CNBD), resulting in reduced IKr channel activity. The lengthened repolarization duration and QT prolongation observed in patients with LQTS can be attributed to the functional impairment caused by this mutation.

We have discovered that the processing of the mutant protein can be corrected through temperature and drug treatment, as demonstrated in both single and double transfections. Previous research has indicated that mutations in the Per-Arnt-Sim domain (PASD) and cyclic nucleotide-binding domain (CNBD) regions can also be corrected through similar temperature and drug interventions [7]. These treatments potentially stabilize the protein conformation, facilitating proper trafficking of the channel protein [8]. It is important to note that some drugs commonly used for correction, such as E4031 and cisapride, carry a risk of QT prolongation and are contraindicated. As an alternative, nicorandil may be considered due to its potential efficacy. The research by Zhou (1999) supports our findings, showing that certain class 2 LQT2 mutations can be improved by culturing cells with an IKr blocker. The effectiveness of drug correction may vary depending on the specific mutation site [9]. Our study suggests that mutations located outside the PASD, pore domain, and CNBD regions can also be corrected through pharmacological interventions.

The mechanism underlying the G418-induced increase in IKr activity is believed to involve the improvement of mRNA translation by bypassing premature termination codons. Previous studies [6,8,10] have suggested that G418, an aminoglycoside antibiotic, can suppress the premature termination of protein synthesis caused by certain mutations. In support of this mechanism, Yu et al. (2014) conducted a study and found that the effectiveness of gentamicin rescue, a similar aminoglycoside antibiotic, was influenced by the proximity of the nonsense mutation site to the N-terminus of the hERG protein [10]. They observed that mutations closer to the N-terminus had a weaker response to gentamicin rescue, which suggests the involvement of NMD mechanism. NMD is a cellular surveillance pathway that targets and degrades mRNAs carrying premature termination codons, thus reducing the expression of potentially harmful truncated proteins. Therefore, in the context of our study, it is plausible that G418 treatment improves the functional expression of the S981fs mutant by bypassing the premature termination codon and allowing for increased translation of the full-length protein. This provides a potential explanation for the observed increase in IKr activity upon G418 treatment in cells expressing the WT*S981fs heterozygotes. Notably, our study indicates that G418 treatment significantly increased IKr current activity only in cells with co-transfection of the wild-type and S981fs alleles (WT/S981fs), while the effect was not significant in cells transfected with S981fs alone. Furthermore, we observed that G418 treatment also increased the expression of wild-type full-length protein. Similar observations have been reported in previous studies, where G418 was found to increase the levels of wild-type proteins [11]. These results suggest that the effect of G418 may be attributed to enhanced expression and translation of the wild-type allele, compensating for the effects of the mutant allele, rather than directly bypassing the stop codon of the mutant allele. It is important to acknowledge that our study relied on cDNAs, limiting our ability to validate the effects of NMD. Therefore, further investigation is needed to elucidate the mechanisms underlying the observed effects of G418.

Overall, our study highlights the utility of WES as a powerful tool for identifying genetic variants associated with inherited disorders like LQTS. WES allows for simultaneous sequencing of numerous genes, offering a comprehensive and cost-effective approach to uncovering the genetic basis of disease. Once the causative variant is identified in an LQTS patient, targeted sequencing of the specific variant can be performed for cascade screening of at-risk family members. For instance, in this study, the proband’s two siblings with a history of seizures could undergo this screening. This approach enables early identification of individuals who may be susceptible to LQTS, facilitating proactive management and prevention of potentially life-threatening arrhythmias.

## 4. Materials and Methods

### 4.1. Study Population

The proband in this study was a 30-year-old female who had a history of seizures since the age of 15. Her seizure episodes occurred during sleep and while talking on the telephone. Her two elder siblings also had a history of seizures. These seizure episodes were initially misinterpreted as epilepsy. However, during her recent visit to the emergency room, it became evident that her seizure attacks were actually episodes of ventricular tachycardia, as documented by the medical team. The proband experienced an episode of ventricular tachycardia with loss of consciousness, requiring immediate defibrillation in the emergency department (Figure 1B). After defibrillation, her ECG showed sinus rhythm with broad T waves and T wave inversion at lateral leads, along with a prolonged corrected QT value of 646 ms (Figure 1C). Echocardiography and coronary angiography did not reveal any abnormalities, and she subsequently underwent implantable cardioverter defibrillator implantation.

### 4.2. Exome Sequencing and Variant Calling

Genomic DNA was extracted from blood samples using a Puregene DNA Isolation Kit (Qiagen, Minneapolis, MN, USA). Whole-exome sequencing was performed on high-quality genomic DNA (100 ng) using the Ion AmpliSeq Exome RDY plates and Ion Proton platform (Ion Torrent, Carlsbad, CA, USA), as previously described [12]. The raw sequencing data were processed using the Torrent sequence-generation algorithm, and single-nucleotide variants and insertion/deletion variants were called using the Torrent Variant Caller (TVC). The variant data were aligned and mapped to the NCBI reference genome (GRCH37/h19) and further analyzed and filtered using Ion Reporter (IR) software (Ion Torrent, Carlsbad, CA, USA). Sanger sequencing was performed to confirm the identified variant.

### 4.3. Construction of GFP-HERG and Site-Directed Mutagenesis

Constructs with and without green fluorescent protein (GFP) fused to C-terminus of HERG (RG215928 and OHu23279) were purchased from OriGene Technologies Inc. (Rockville, MD, USA) and GeneScript Inc. (Piscataway, NJ, USA), respectively. The p.S981fs-GFT and p.S981fs mutant constructs were generated using the QuickChange Site-Directed Mutagenesis system (Q5^®^Site-directed mutagenesis kit, New England Biolabs, Ipswich, MA, USA). Direct DNA sequencing was performed to verify all the constructs.

### 4.4. Culture and Transfection of HEK293T Cells

HEK293T cells were cultured in Dulbecco’s Modified Eagle’s Medium (Gibco, BRL Life Technologies, Inc., Rockville, MD, USA) supplemented with 10% fetal bovine serum and antibiotics at 37 °C and 5% CO_2_. For transfection, 3 µg of cDNA was used for mono-transfection experiments, while 1.5µg of wild-type (WT) and S981fs cDNA were co-transfected using Lipofectamine 2000 (Invitrogen, Carlsbad, CA, USA). After transfection, the cells were either trypsinized for patch clamp experiments or scraped for Western blot analysis at 24 or 48 h post-transfection.

To investigate the correction of deficient Kv11.1 function, specific blockers and openers were added to the culture media for 24 h prior to the experiments. These included E4031, a blocker of delayed rectifier current (Ikr); cisapride, a potassium channel blocker; nicorandil, an ATP-sensitive potassium-channel opener; and G-418, an antibiotic.

### 4.5. Expression Analysis

To investigate the expression of mutant HERG proteins on cell membranes, transfected HEK293T cells were immunostained with an anti-HERG antibody against the extracellular peptide (Sigma K0640; Sigma-Aldrich, St Louis, MO, USA) and incubated with FITC-conjugated secondary antibody. Confocal images were obtained and analyzed using a confocal laser-scanning microscope (TCS SP2, Leica, Wetzlar, Germany).

### 4.6. Western Blot and Co-Immunoprecipitation

The transfected cells were lysed in a lysis buffer containing 5 mM EDTA, 250 mM NaCl, 50 mM HEPES, and 0.1% NP40. The lysates were subjected to SDS-PAGE, transferred to PVDF membrane, and detected by Western blot using anti-HERG antibodies (F-12 from Santa Cruz, Delaware Avenue, CA, USA and APC016 from Allomone labs, Jerusalem, Israel). Co-immunoprecipitation experiments were performed using anti-GFP antibody (Invitrogen, Carlsbad, CA, USA) and protein A/G plus (Santa Cruz, Delaware Avenue, CA, USA). The immunoprecipitated proteins were released from the beads and analyzed by sequential Western blot analysis.

### 4.7. Patch Clamp Experiments of HEK293T Cells

Kv11.1 current (IKv11.1) was recorded using the whole-cell patch-clamp technique. The extracellular bath solution used during the experiments contained the following concentrations (in mmol/L): 137 NaCl, 5.4 KCl, 2 CaCl_2_, 1.1 MgCl_2_, 0.33 NaH_2_PO_4_, 5.6 glucose, and 10 HEPES. The pH of the bath solution was adjusted to 7.4 with NaOH. The intracellular pipette solution contained the following concentrations (in mmol/L): 120 K-Aspartate, 20 KCl, 5 EGTA, 5 MgATP, 10 Hepes, and 5 Na Creatine P. The pH of the pipette solution was adjusted to 7.2 with KOH. The cells were held at a holding potential of −80 mV during the experiments. All voltage clamp experiments were conducted at a temperature of 22 °C to 23 °C. Current densities were calculated by dividing the peak current by the cell membrane capacitance. Data acquisition was performed using pCLAMP 8.0 software on an IBM compatible computer equipped with a 16-bit Digidata 1320A interface (Molecular Devices, Sunnyvale, CA, USA). The acquired data were analyzed using Clampfit 10.3 software (Molecular Devices, Sunnyvale, CA, USA).

### 4.8. Statistical Analysis

The data are presented as mean ± SEM. Statistical analysis was performed using Student’s *t*-test and ANOVA coupled with Tukey’s test. A *p*-value less than 0.05 was considered significant.

## 5. Conclusions

Our study identified a novel frameshift mutation (S981fs) in the KCNH2 gene in a patient with LQT2. This mutation resulted in reduced cell membrane expression of the hERG channel and decreased IKr currents, likely due to impaired protein synthesis and trafficking. Importantly, we found that treatment with the aminoglycoside antibiotic G418 significantly increased the hERG current in cells expressing the WT/S981fs heterozygote, suggesting its potential as a therapeutic intervention for LQT2.

## Data Availability

The data presented in this study are available upon request from the corresponding author.
